# Galectin-1 Cooperates with Yersinia Outer Protein (Yop) P to Thwart Protective Immunity by Repressing Nitric Oxide Production

**DOI:** 10.3390/biom11111636

**Published:** 2021-11-04

**Authors:** Brenda Lucila Jofre, Ricardo Javier Eliçabe, Juan Eduardo Silva, Juan Manuel Pérez Sáez, Maria Daniela Paez, Eduardo Callegari, Karina Valeria Mariño, María Silvia Di Genaro, Gabriel Adrián Rabinovich, Roberto Carlos Davicino

**Affiliations:** 1 División de Inmunología, Facultad de Química, Bioquímica y Farmacia, Universidad Nacional de San Luis, San Luis CP5700, Argentina; brendalucila.jofre@gmail.com (B.L.J.); javielicabe@gmail.com (R.J.E.); jesilva9@hotmail.com (J.E.S.); sdigena@gmail.com (M.S.D.G.); 2 Instituto Multidisciplinario de Investigaciones Biológicas (IMIBIO), Consejo Nacional de Investigaciones Científicas y Técnicas (CONICET), San Luis C5700, Argentina; 3 Laboratorio de Glicomedicina, Instituto de Biología y Medicina Experimental (IBYME), Consejo Nacional de Investigaciones Científicas y Técnicas (IBYME-CONICET), Buenos Aires C1428ADN, Argentina; juanmanuelperezsaez@gmail.com (J.M.P.S.); gabyrabi@gmail.com (G.A.R.); 4 Division of Basic Biomedical Sciences, Sanford School of Medicine, University of South Dakota, Vermillion, SD 66544, USA; Daniela.Paez@usd.edu (M.D.P.); Eduardo.Callegari@usd.edu (E.C.); 5 Laboratorio de Glicómica Funcional y Molecular, Instituto de Biología y Medicina Experimental, Consejo Nacional de Investigaciones Científicas y Técnicas (IBYME-CONICET), Buenos Aires C1428ADN, Argentina; kmarino@ibyme.conicet.gov.ar; 6 Departamento de Química Biológica, Facultad de Ciencias Exactas y Naturales, Universidad de Buenos Aires, Buenos Aires C1428, Argentina; 7 Roberto Davicino, División de Inmunología, Facultad de Química, Bioquímica y Farmacia, Universidad Nacional de San Luis, Ejercito de los Andes 950, San Luis CP5700, Argentina

**Keywords:** *Yersinia enterocolitica*, YopP, Galectin-1, nitric oxide, macrophages

## Abstract

*Yersinia enterocolitica* (Ye) inserts outer proteins (Yops) into cytoplasm to infect host cells. However, in spite of considerable progress, the mechanisms implicated in this process, including the association of Yops with host proteins, remain unclear. Here, we evaluated the functional role of Galectin-1 (Gal1), an endogenous β-galactoside-binding protein, in modulating Yop interactions with host cells. Our results showed that Gal1 binds to Yops in a carbohydrate-dependent manner. Interestingly, Gal1 binding to Yops protects these virulence factors from trypsin digestion. Given that early control of Ye infection involves activation of macrophages, we evaluated the role of Gal1 and YopP in the modulation of macrophage function. Although Gal1 and YopP did not influence production of superoxide anion and/or TNF by Ye-infected macrophages, they coordinately inhibited nitric oxide (NO) production. Notably, recombinant Gal1 (rGal1) did not rescue NO increase observed in *Lgals1*^−/−^ macrophages infected with the YopP mutant Ye ∆*yopP*. Whereas NO induced apoptosis in macrophages, no significant differences in cell death were detected between Gal1-deficient macrophages infected with Ye ∆*yopP*, and WT macrophages infected with Ye wt. Strikingly, increased NO production was found in WT macrophages treated with MAPK inhibitors and infected with Ye wt. Finally, rGal1 administration did not reverse the protective effect in Peyer Patches (PPs) of *Lgals1^−/−^* mice infected with Ye ∆*yopP*. Our study reveals a cooperative role of YopP and endogenous Gal1 during Ye infection.

## 1. Introduction

*Yersinia enterocolitica* (Ye), *Yersinia pseudotuberculosis*, and *Yersinia pestis* are the three human pathogenic bacteria in the genus *Yersinia* [1]. Ye causes food-borne self-limiting severe diarrhea, enteritis, and mesenteric lymphadenitis. In addition to gastrointestinal effects, Ye gradually spreads across the body, causing symptoms in the liver and spleen [2,3]. Ye uses a type III protein secretion machinery to deliver into host cells bacterial effector proteins encoded in the 70-kb *Yersinia* virulence plasmid (pYV). This plasmid includes a set of six effector Yersinia outer proteins (Yops): YopE, YopH, YopM, YopO/YpkA, YopP/YopJ, YopT [4]. YopH counteracts phagocytosis and T-cell activation [5,6], while YopE, YopT, and YopO disrupt actin cytoskeleton [7,8]. In addition, YopP/J inhibits nuclear factor kappa B (NF-kB) signaling, suppresses pro-inflammatory cytokines, modulates antigen uptake, and induces apoptosis in macrophages and dendritic cells [9,10,11,12,13]. Moreover, YopP inhibits the activation of MAPKs inactivating c-Jun-N-terminal kinase (JNK), p38, and extracellular signal-regulated 1/2 kinase (ERK1/2) [14,15,16]. In this context, YopP can interact directly or indirectly with specific kinases, acting as a “poison kinase” [16]. In this regard, YopP is an acetyltransferase, which uses acetyl-coenzymeA(acetyl-CoA) as a cofactor to acetylate critical serine and threonine residues in the activation loop of MAPKKs and IKK-I3 [12,17]. Surprisingly, MAPK as well as NF-kB, are constrained in scaffolds and the recruitment of YopP to such a scaffold would allow faster inhibition of signaling events compared to a free diffusion of YopP in the cell [18]. In addition, YopP is activated by the host cell factor inositolhexakisphosphate (IP6), which could also explain how YopP is kept in a quiescent state in the bacterium, since bacteria lack the capacity to synthesize IP6 [19]. In activated macrophages however, *Yersiniae* cause pyroptosis, a cell death program independent of YopP, which involves inflammasome activation and processing of caspase-1, release of pro-inflammatory cytokines IL-1β and IL-18, and eventually lysis of macrophages and release of pro-inflammatory intracellular content [20,21]. The prevention of pyroptosis and suppression of inflammatory response by YopP could be crucial for *Yersiniae* ability to colonize the Peyer’s patches without an initial immune response [22,23,24]. In this context, the early control of Ye infection is mediated by innate immune mechanisms, involving natural killer (NK) cells, neutrophils and macrophages [25,26,27].

Interestingly, M1 and M2 macrophages refer to the two extremes of a spectrum of potential macrophage activation states; however the term M2 has been traditionally used for any macrophage activation states other than M1. The use of M2 as a generic term for macrophage activation is justified by the fact that they share a number of functional characteristics and are involved in immunoregulation and tissue remodeling. In this regard, threesubclassesof M2 macrophages have been identified: M2a, triggered by IL-4 or IL-13; M2b, induced by exposure to Toll-like receptor (TLR) agonists and IL-1R; and M2c, induced by IL-10 and glucocorticoids [28]. On the other hand, M1 macrophage activation is defined by high production of toxic intermediates, such as reactive oxygen species (ROS) and NO [28]. However, few reports are available on the role of NO in Ye infection [29,30]. We have previously shown increased NO synthesis and enhanced expression of inducible nitric oxide synthase (iNOS) in response to Ye antigens in macrophages from mice lacking the tumor necrosis factor receptor p55 (TNFRp55) [31]. These results suggested a role of TNFRp55 and NO in modulating macrophage functions after Ye infection. In addition, we have shown that Ye infection induces local and systemic up-regulation of Galectin-1 (Gal1), an endogenous immunomodulatory lectin, which blunts NO synthesis and limits bacterial clearance [32].

Through binding to β-galactoside-containing glycoconjugates, Gal1 triggers different biological processes including those operating during innate and adaptive immune responses, as well as those involving host-pathogen interactions. Gal1, as well as other members of this lectin family, can cross-link glycosylated receptors, including: the T cell receptor (TCR); pre-B cell receptor (pre-BCR) and CD45, facilitating their cell surface retention and modulating signaling thresholds [33,34]. In this regard, it has been demonstrated that glycan-binding proteins may serve as a bridge that regulates bacterial infection, internalization and immunity [35,36].

Thus, given the emerging roles of Gal1 in infection [33,37,38,39,40,41,42,43] and based on our previous results showing that *Y. enterocolitica* induced a YopP dependent positive regulation of Gal1 [32], we hypothesized that Yops could interact with Gal1 and modulate the course of Ye infection. In the present work we studied the interactions between Yops and Gal1, focusing on the role of the Ye virulence factor YopP in shaping the course of early innate immune response upon Ye infection.

## 2. Materials and Methods

### 2.1. Bacterial Culture and Purification of Yops

Infection was performed with Ye serotype 0:8 (pYV+, WA-314) (Ye wt) or with Ye WA-314 deficient in YopP (pYV+, WA-C pYVNalrKanr) (Ye ∆*yopP*) [44], kindly provided by Ingo Autenrieth (Tuebingen, Germany). Bacteria were cultured as previously described [45], diluted 1:20, and incubated at 37 °C with agitation for 2 h (180 rpm). The addition of EGTA (5 mM) for Ca^2+^ chelation, MgCl_2_ (15 mM), and glucose (0.2%) induced Yops expression and secretion. Bacteria were grown at 37 °C for 2 to 3 h and centrifuged (10,000× *g* for 15 min), and proteins were precipitated from culture supernatants with trichloroacetic acid (TCA) as previously described [46].

### 2.2. Mice and Infection

C57BL/6 Gal1 knockout (*Lgals1*^−/−^) mice were kindly provided by F. Poirier (Institute Jacques Monod, Paris, France). C57BL/6 wild-type (WT) mice were purchased from the Animal Facilities of the National University of La Plata, La Plata, Argentina. Breeding colonies were established at the animal facilities of the National University of San Luis (San Luis, Argentina). Mice were housed in a cabinet (Ehret, Emmendingen, Germany) and given ad libitum sterile food and water. Male mice (6–8 wk-old) were used for all the experiments. The Animal Care and Use Committee of the National University of San Luis, Argentina, approved the experimental protocols (Protocol Number: B226/16).

Mice were starved for 2 h before being inoculated orogastrically with 5 × 10^8^ bacteria in 0.2 mL sterile phosphate-buffered saline solution (pH 7.4) using a gastric tube. PBS was given to the control mice. Serial dilutions of the inoculated suspension were plated on Trypticase soy agar (Britania, Buenos Aires, Argentina) to monitor the real number of inoculated bacteria.

The PPs were removed in aseptic conditions and homogenized in PBS. Then, on MacConkey-Igarsan agar, duplicates of 50 µL of serial dilutions of PPs homogenates were plated (Britania, Buenos Aires, Argentina). After 48-h incubation period at 27 °C, colony-forming units (CFU) were counted. The limit of detectable CFU was 25 (log_10_25 = 1.4) [47].

### 2.3. Stimulation of Peritoneal Macrophages

*Lgals1^−/−^* and WT resident peritoneal macrophages were isolated from mice of both genotypes using 10 mLof sterile pyrogen-free saline solution, centrifuged twice at 200× *g* for 10 min at 4 °C, and re-suspended in DMEM supplemented with 10% heat-inactivated fetal bovine serum (FBS) (Natocor, Córdoba, Argentina), 5 mM L-glutamine, 50 µM 2-ME, 100 IU/mL penicillin, 100 µg/mL streptomycin, and 50 µg/mL gentamicin (Thermo Fisher Scientific, Waltham, Massachusetts, EEUU).This cellular suspension was seeded onto a 24-well culture plate (Costar, Tecnolab, Buenos Aires, Argentina) at 2 × 10^6^ cells per well. After 24 h of incubation at 37 °C in a 5% CO_2_ atmosphere, adherent cells were washed three times with saline and incubated for 1 h at 37 °C in a 5% CO_2_ atmosphere with or without Ye wt or Ye ∆*yopP* (multiplicity of infection, moi: 10:1) in the absence or presence of 5 μM ERK1/2 inhibitor (PD98059) or p38 inhibitor (SB203580) (Calbiochem, San Diego, CA, USA). To eliminate extracellular bacteria, 0.1 g/mL of gentamicin was added. Cells were incubated overnight, and culture supernatants were collected [32].

### 2.4. NO and Urea Determination

The Griess reaction assay was used to measure nitrite synthesis in macrophage culture supernatants obtained 12 h after Ye infection [32]. In a 96-well flat-bottom plate, 100 µL of culture supernatant was mixed with 100 µL of Griess reagent and incubated for 10 min at room temperature. Absorbance at 550 nm was determined in a plate reader (Bio-Rad, New York, NY, USA). In addition, urea was measured in macrophage culture supernatants using the Urea Color 2R package (Wiener, Rosario, Argentina), according to the manufacturer’s instructions.

### 2.5. Apoptosis Assays

Macrophages isolated from WT or *Lgals1^−/−^* mice were infected with Ye wt or with Ye ∆*yopP* and incubated in a 5% CO_2_ atmosphere. Dimethyl sulfoxide (DMSO) (Sigma, St. Louis, MO, USA) was used as a positive control for apoptosis. Cells (1 × 10^6^) were suspended in binding buffer (10 mM HEPES pH 7.4, 140 mM NaCl, 2.5 mM CaCl_2_) after being washed twice with PBS. Macrophages were incubated for 15 min at room temperature in the dark with Annexin V-FITC (Sigma, St. Louis, MO, USA). The cells were washed and re-suspended in 500 µL of binding buffer. Finally, macrophages were stained with propidium iodide (PI) (Sigma, St. Louis, MO, USA) and analyzed by flow cytometry using a FACSCalibur cytometer (Becton, Dickinson and Company, Franklin Lakes, NJ, USA).

### 2.6. Preparation and Purification of RGal1

Recombinant Gal1 (rGal1) was produced and purified as previously described [48]. Briefly, *LGALS1* gene was cloned into a pET-3a (+) vector for bacterial expression between the NdeI and BamHI specific recognition sites. The plasmid was first amplified in DH5α *E. coli* and subsequently used for transformation of *E. coli* C41 (DE3) pLysS. The resulting protein was purified by affinity chromatography on a lactosyl-Sepharose resin. Purified Gal1 was dialyzed against PBS (pH 6–9) for 6 h, three times and then subjected to a Polymixin B affinity resin to remove endotoxins from protein solution. Protein was measured by the Pierce BCA Protein Assay Kit (Thermo Fisher, Carlsbad, CA, USA), according to the manufacturer protocol. The recombinant protein was sterilized by passage through a 0.22-μm syringe filter, adjusted to 10 mg/mL in PBS and stored as frozen aliquots until used.

### 2.7. ELISA Assays

TNF and IL-10 were determined in supernatants of infected WT or *Lgals1^−/−^* macrophages using capture ELISA kits (eBioscience, San Diego, CA, USA) according to the manufacturer’s instructions. YopP was determined using a modified ELISA protocol described by Chatzipanagiotou et al., 2001 [49]. Briefly, Yops were prepared from Ye wt or Ye ∆*yopP*. ELISA plates (Corning, Kennebunk, ME, USA) were coated with Yops antigens (10 µg/well) and the binding of rGal1 (10 mg/mL) was detected using rabbit anti-Gal1 antibodies (1/1000). The absorbance was read at 450 nm using a plate reader (Bio-Rad, New York, NY, USA).

### 2.8. Oxidative Burst Assay

For this assay, we used a protocol described by Schopf et al. (1984) [50] and ROS products were evaluated by the reduction of nitro blue tetrazolium (NBT) (Sigma, St. Louis, MO, USA) to formazan. In all these assays, WT or *Lgals1^−/−^* macrophages were infected with Ye wt or with Ye ∆*yopP*, then, 300 μL of NBT was added and the reaction was stopped with 1N HCl (Tetrahedron, Buenos Aires, Argentina). Dioxane (Dorwill, Buenos Aires, Argentina) was used to obtain formazan, and the absorbance was determined in a microplate reader at 525 nm (Bio-Rad).

### 2.9. In Vivo and In Vitro Supplementation of RGal1

For in vivo phenotype-rescuing studies, four animal groups were used: groups 1 and 2 were *Lgals1^−/−^* mice injected i.p. with rGal1 (3.2 mg/kg) or vehicle control daily for 5 days after Ye wt infection [32]; groups 3 and 4 were *Lgals1^−/−^* mice injected i.p. with rGal1 (3.2 mg/kg) or vehicle control daily for 5 days after Ye ∆*yopP* infection [32]. Mice were killed five days after infection, and CFU were counted in PPs homogenates as mentioned previously.

For in vitro rescue experiments, peritoneal macrophages from *Lgals1^−/−^* mice were pretreated with 4 µg/mL rGal1 for 2 has previously described [51], and then infected with Ye wt or Ye ∆*yopP* as outlined above. Then, supernatants were obtained and tested for NO and urea production.

### 2.10. Analysis of Yops-Gal1 Interactions by SDS-PAGE and Western Blot

Briefly, 25 μL of Yops were added to each well and resolved by 12% SDS-PAGE. Subsequently, bands were transferred onto a PVDF membrane (Bio-Rad, New York, NY, USA), which was then blocked with 3% bovine serum albumin (BSA) and incubated for 18 h at 4 °C in agitation with human rGal1 (6 µg/mL) or with rGal1 plus 30 mM of lactose as a disaccharide competitor. Finally, an in-house generated polyclonal anti-Gal1 antibody [52] was diluted 1:1000 and the reaction was revealed using chemiluminescence detection kit (Amersham Biosciences, London, UK). To evaluate the importance of glycans in Yops-Gal1 interaction, glycan oxidation was achieved by treatment with 10 mM NaIO_4_ as previously described [53].

### 2.11. Lectin Blotting

Yops were run in 10% SDS-PAGE, and transferred onto 0.45-μm PVDF membranes (Bio-Rad). Membranes were then blocked with 3% bovine serum albumin (BSA) and strips were probed with the biotinylated lectins listed in Table 1,as previously described [54]. Lectin binding was visualized using horseradish rabbit peroxidase (HRP)-conjugated streptavidin (Sigma) with and C-DiGit^®^ Blot Scanner (LI-COR Biosciences, Lincoln, NE, USA).

### 2.12. Yops Proteolysis Using Trypsin Digestion

Yops obtained from Ye wt were incubated with or without rGal1 overnight. Samples were subsequently digested with trypsin (200 μg/mL) (Sigma) following the protocol described by Shevchenko et al. (2006) [57]. Digestion products were subjected to separation in denaturing SDS-PAGE and Yops-rGal1 association was evaluated by Western blot using anti-Gal1 antibodies (1:1000). The reaction was revealed by chemiluminescence using the C-DiGit^®^ Blot Scanner (LI-COR Biosciences, EEUU).

### 2.13. Schiff Staining

Briefly, Yops were added to each well and resolved by SDS-PAGE, and subsequently the gel was immersed in 12.5% trichloroacetic acid overnight and then placed in 1% periodic acid. Finally, the gel was incubated with Schiff’s reagent in the dark for 1 h and washed with 0.5% of sodium metabisulphite three times for 10 min followed by distilled water [58].

### 2.14. Flow Cytometry

Ye (2 × 10^8^ CFU) were fixed in 3% paraformaldehyde for 2 h at room temperature, washed three times in phosphate-buffered saline (PBS), and stored at −80 °C in PBS containing 15% of glycerol. To determine galectin binding, 2 × 10^7^ fixed bacteria were incubated with label free rGal1 as described [59] at a final concentration of 3.3 mM (100 µg/mL) for 1 h at 37 °C. After two washes with PBS/Tween 0.1%, Gal1 binding was detected by incubation with a rabbit anti-human Gal1 antibody for 45 min at 4 °C. Cells were then washed twice in 0.1% PBS-Tween, next, resuspended in 50 μL of PBS with a polyclonal anti-rabbit FITC-conjugate antibody (1/200), and incubated for 30 min on ice. Galectin binding was determined using a flow cytometer (FACSCalibur), and at least 4 × 10^4^ events were recorded. Gal1 binding was evaluated by calculating the Fluorescence Medium Index [% positive gated bacteria multiplied by the geometric mean fluorescence] [60].

### 2.15. Mass Spectrometry

Proteins were separated through 1D-SDS-PAGE on 10% of polyacrylamide gels. Next, gels were stained with 0.1% of Coomassie R-250. Selected bands were excised from gels and sent to the Center for Chemical and Biological Studies by Mass Spectrometry (CEQUIBIEM), Faculty of Exact and Natural Sciences, University of Buenos Aires, where protein identification analysis was performed. Briefly, bands were de-stained with 50 mM of ammonium bicarbonate/acetonitrile (50/50% *v*/*v*), reduced with DTT, followed by alkylation with iodoacetamide. Trypsin sequencing grade was used for in-gel digestion (Promega, Madison, WI, USA). The tryptic digested peptides were resuspended in 0.1% formic acid in water, injected into Easy nLC 1000 (Thermo Scientific), and analyzed by tandem mass spectrometry using a QExactive Orbitrap mass spectrometer (Thermo Scientific) [61].

### 2.16. Bioinformatics Analysis

The Mascot Generic Format (mgf) files were extracted from a RAW files using Mascot Distiller program v2.6.2.0 (www.matrixscience.com, original search: 12 December 2019 and corroborated through a most recently search on 11 January 2021), and searched against Yop 20191212 in house customized database (accession WP_010891200.1 from RefSeq, NCBI, 1 sequence, 288 residues) using Mascot server 2.6.2 (www.matrixscience.com, similar date than Distiller) local license. MASCOT server v2.6.2 in MS/MS ion search mode was applied to conduct peptide matches (peptide masses and sequence tags) and protein searches using the database mentioned previously. The following parameters were established for search: carbamidomethyl (C) on cysteine was set as fixed, and variable modifications included asparagines and glutamine deamidation, and methionine oxidation, respectively. Only two missed cleavages were allowed. Monoisotopic masses were counted. The precursor peptide mass tolerance was set at 20 ppm. Fragment mass tolerance was 0.02 Da and the ion score or expected cutoff was set at 5. The MS/MS spectra were searched with MASCOT using a 95% confidence interval (CI%) threshold (*p* < 0.05), while minimum score of 14 was used for peptide identification. Furthermore, the error tolerance mode was set up at MASCOT search to corroborate potential peptides unidentified during the first search.

### 2.17. Western Blot Analysis of INOS Expression

WT or *Lgals1^−/−^* macrophages were infected with Ye wt or Ye ∆*yopP*. Cell lysates (40 μg of protein/lane) were size fractionated in 12% SDS-polyacrylamide gel electrophoresis and transferred to a PVDF membrane. Membranes were incubated for 90 min in Tris buffered saline (TBS, pH 7.5)-3% milk and then overnight with a 1:200 rabbit antibody against iNOS (Santa Cruz Biotechnology Inc., Santa Cruz, CA, USA). Membranes were washed with TBS- 0.05% Tween 20 and incubated with a 1:1000 horseradish peroxidase-conjugated goat anti-rabbit IgG (Sigma, CA, USA). Immunodetection was performed using chemiluminescence, following the protocol provided by the manufacturer. The immunoreactive protein bands were analyzed using the ImageJ software.

### 2.18. Statistical Analysis

The Mann-Whitney U test or one-way ANOVA with the Dunnett multiple-comparison test were used to determine if the differences between the groups were significant. Results are expressed as the mean ± SEM. All statistical analyses were carried out using Prism version 5.0 (GraphPad, La Jolla, CA, USA). *p* values < 0.05 were considered statistically significant.

## 3. Results

### 3.1. Galectin-1 Binds to YopP in Y. enterocolitica

**Figure 1:**
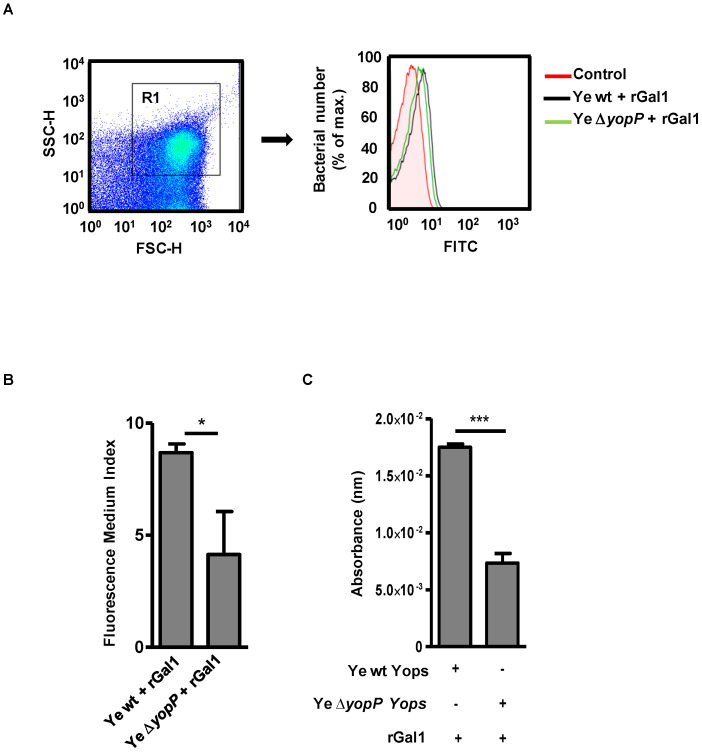
Galectin-1 Binds to Ye YopP.Binding of rGal1 to Ye wt (black) or to Ye ∆*yopP* (green) is shown. Control (Ye without rGal1) is shown in red. Binding was analyzed by flow cytometry usinga FITC-conjugated anti-Gal1 antibody (**A**). Representative flow cytometry analysis of two independent experiments, showing the gate in Region 1 (R1) and the histogram expressing the number of FITC-positive bacteria. (**B**) Binding is expressed as the Fluorescence Medium Index. (**C**) ELISA plates were coated with Yops from Ye wt or Ye ∆*yopP* (10 µg/well) obtained under the same conditions and incubated with 10 mg/mL of rGal1. Gal1 binding was detected using an anti-Gal1 rabbit polyclonal antibody (1/1000). Data are the mean ± SEM of three independent experiments (**B**,**C**). * *p* < 0.05, *** *p* < 0.001.

Previous findings demonstrated the presence of Yops in the membrane fraction of Ye [4]. To explore whether Gal1 can bind to Ye surface proteins, we performed flow cytometry and ELISA using Gal1 as a probe, either with Ye wt or Ye ∆*yopP*, a genetically modified bacteria devoid of YopP (Figure 1).

We found a significant decrease in Gal1 binding to Ye ∆*yopP* compared to Ye wt (Figure 1A,B; *p* < 0.05, Figure 1C, *p* < 0.001). Given that secretion of YopP by Ye is significantly lower than secretion of other Yops [62], these results suggest that YopP could mediate Gal1 binding to Ye, although other mediators maybe also contribute to this effect.

### 3.2. Galectin-1 Recognizes Yops in a Carbohydrate-Dependent Manner

**Figure 2:**
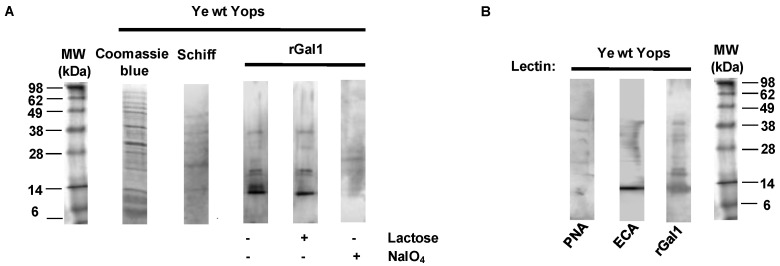
Virulence factors secreted by *Y. enterocolitica* are glycosylated and exhibit Gal1-permissive glycoepitopes. Electrophoresis was performed using an SDS-polyacrylamide gel (25 μL per well of Ye wt Yops; 20–40 μg/well). Subsequently, gels were (**A**) treated with rGal1 (6 µg/mL), rGal1 and lactose (30 mM) or NaIO_4_ (10 mM) or (**B**) incubated with biotinylated *Peanut agglutinin* (PNA) or *Erythrina cristagalli lectin* (ECA), capable of recognizing disaccharides with lactose-derived structures. Detection was performed by Coomassie blue staining, Schiff staining (**A**) or revealed by chemiluminescence (**B**). Data are representative of two independent experiments.

To evaluate potential Gal1 ligands in Ye and given the glycan-binding activity of this protein, we studied whether Gal1-Ye interactions are mediated by specific glycans. Previous studies demonstrated that Ye spp. presents an alternative bacterial pathway, mediated by a cytoplasmic N-glycosyltransferase, a homolog of *Actinobacillus pleuropneumoniae* HMW1C-Like glycosyltransferase (ApHMWC1LGT). This enzyme uses nucleotide-activated monosaccharides as donors to modify asparagine residues, and transfer glucose and galactose with NX(S/T) as the acceptor sequon [63]. A secondary O-glycosylation activity was described for ApHMWC1LGT transferring a donor sugar to an acceptor sugar, forming di-hexoses on glycoproteins [64]. However, no data are available regarding the O-glycosylation pathway in this bacterium. We found, by means of a classical Schiff staining, that Yops are glycosylated (Figure 2A). In order to evaluate glycan-dependent binding of Gal1 to Yops, these glycoproteins were separated by SDS-PAGE and incubated with rGal1 in the absence or presence of lactose (30 mM) as a competitive carbohydrate inhibitor of Gal1 binding activity (Figure 2A). Gal1-glycan interactions were inhibited by lactose at the level of protein bands corresponding to 14, 25 and 35 kDa, suggesting carbohydrate-dependent binding of this lectin to secreted proteins of Ye. Thus, periodate treatment (which induces glycan oxidation) impaired Gal1 binding, indicating that Gal1-Yop interactions are mediated by specific glycosylated structures (Figure 2A).

Based on these findings, we next investigated the presence of Yops glyco-epitopes in electrophoretically-resolved protein bands, using biotinylated plant lectins able to recognize glycan structures permissive for Gal1 binding. Lectin blotting revealed the presence of 14–38 kDa bands that bound to *Erythrina cristagalli* (ECL), a lectin capable of recognizing non-sialylated *N*-acetyllactosamine (LacNAc, Galβ(1–4)GlcNAc) structures. Notably, *peanut agglutinin* (PNA) reactivity was also (albeit faintly) observed, suggesting that Ye glycoproteins may also display glycans with Galβ(1–3) terminal structures(Figure 2B). These results indicate that β-galactoside residues are exposed in Yops and may act as possible glycoepitopes for binding of host Gal1.

### 3.3. Mass Spectrometry-Based Proteomics Analysis of Yops

**Figure 3:**
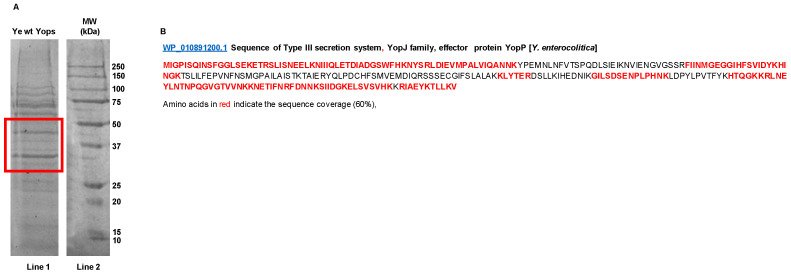
Protein bands derived from Yops selected for MS/MS analysis.Yops were solved in 1D- SDS-PAGE gels and stained with Coomassie Blue G-250 (**A**, Lane 1). Molecular weight markers (MW) are shown in (**A**), Lane 2. The red squares indicate bands subjected to identification through MS/MS analysis. YopP peptides identified (highlighted in red) in the selected bands and protein coverage map of Type III secretion system YopJ family effectors are shown in (**B**).

It has been demonstrated that YopP is a critical virulence factor involved in bacterial immune evasion [65] and Gal1 contributes to Ye-driven immunosuppression [32]. Separation of Yops proteins by 1D-SDS-PAGE and identification of bands using nanoLC-MS/MS analysis revealed selected bands corresponding to molecular weights ranging from 30 to 55 kDa (Figure 3A, red square) with nineteen identified peptides matching YopP, including the N- and C-terminus, and representing a 60% of sequence coverage of the protein (Figure 3B and Table 2).

### 3.4. Gal1 Protects Yops from Protease Degradation

**Figure 4:**
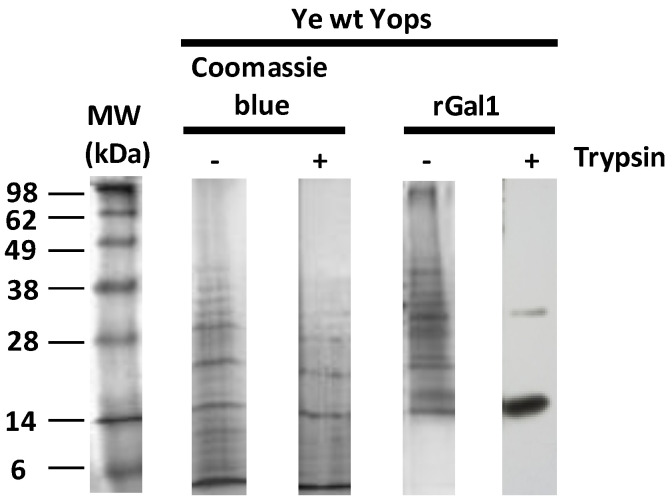
Gal1 protects virulence factors secreted by *Ye* from protease degradation. Electrophoresis was performed in an SDS-polyacrylamide gel (25 μL per well of Ye wt Yops; 20–40 μg/well). Subsequently, gels were treated with trypsin (200 µg/mL). Detection was performed by Coomassie blue staining or revealed by chemiluminescence. Data are representative of two independent experiments.

It has been well established that certain members of the galectin family, such as galectin-4 (Gal4), protect the brush border enzymes in the small intestine of the action of proteinases and lipases through binding to these enzymes [66]. To investigate whether Ye can take advantage of Gal1-glycan interactions and protect Yops from degradation, Yops were incubated with rGal1 and then treated with trypsin (200 µg/mL), separated by SDS-PAGE, and incubated with polyclonal anti-Gal1 antibodies. The results demonstrate the binding of Gal1 to two particular protein bands running in 14 and 35 kDa (Figure 4), suggesting that this lectin might protect these glycoproteins from protease digestion. Since purified Yops could also contain other proteins. Future studies should be conducted to analyze their identity.

### 3.5. Gal1 and YopP Control Y. enterocolitica Infection by Decreasing NO Production

**Figure 5:**
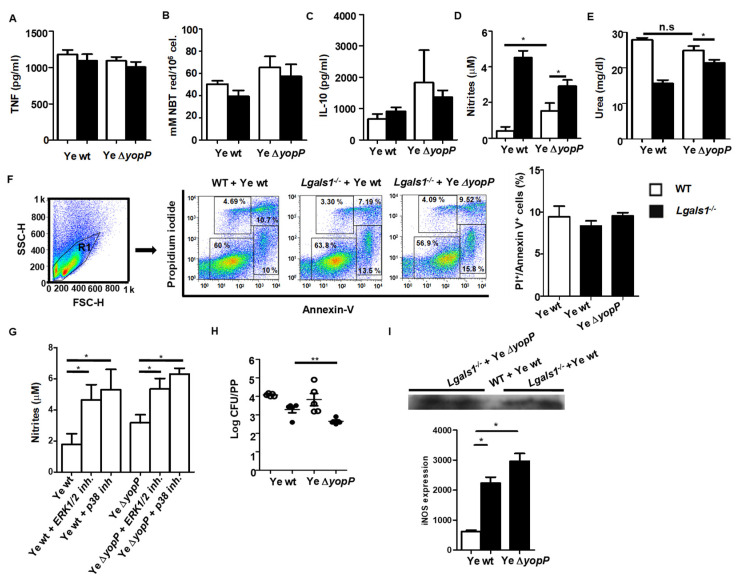
Lack of Gal1 and YopP induces NO Production and Confers Protection Against Ye. WT and *Lgals1^−/−^* macrophages were infected with Ye wt or Ye ∆*yopP* for 1 h. TNF and IL-10 were determined in culture supernatants by ELISA (**A**,**C**). Superoxide anion was determined as described in *Materials and methods* (**B**). NO production was measured in WT and *Lgals1^−/−^* macrophages after Ye Wt or Ye ∆*yopP* infection for 1 h (**D**). Urea was determined in culture supernatant as an indirect assessment of arginase activity (**E**). Macrophages were isolated from *Lgals1^−/−^* or WT mice, infected in vitro with Ye wt or Ye ∆*yopP* for 2 h, stained with annexin-V and propidium iodide and analyzed by flow cytometry. In the gated Region 1 (R1), the percentage of annexin-V^+^ propidium iodide^+^ cells are shown (right panel) (**F**). NO production in macrophages infected in vitro with Ye wt or Ye ∆*yopP* in the absence or presence of ERK1/2 or p38 inhibitors (**G**). CFU were evaluated in PPs of mice infected with Ye wt or with Ye ∆*yopP* after 5 days. Limit of detectable CFU was 25 (log_10_25 = 1.4) (**H**). After infection, macrophage lysates were analyzed by Western blot using specific antibodies against iNOS (**I**). Data are the mean ± SEM of three independent experiments (**A**–**F** right panel and I bottom panel), representative of three independent experiments (**F** left panel and **I** upper panel) or representative of two independent experiments (**H**, *n* = 5 mice per group). * *p* < 0.05, ** *p* < 0.01.

To further address the functional relevance of Gal1-YopP interactions during Ye infection, we first evaluated the impact this endogenous lectin in oxidative burst and inflammatory response. Given that superoxide (O_2_^−^) and tumor necrosis factor (TNF) contribute to innate responses of resident macrophages [67], we evaluated the O_2_^−^ and TNF production by Ye-infected resident macrophages in the presence or absence of Gal1. We found that O_2_^−^ and TNF were not significantly different in *Lgals1^−/−^* macrophages infected with Ye wt or Ye ∆*yopP* compared with macrophages isolated from WT infected mice (Figure 5A,B). Interestingly, in spite of the ability of YopP to inhibit TNF through MAPKs [14], we found no significant difference in TNF production by Ye ∆*yopP*-infected WT macrophages compared to WT macrophages infected with Ye wt (Figure 5A). In this sense, the production of IL-10 was evaluated, given its well-established role in attenuating TNF synthesis [30]. We studied IL-10 production byn WT and *Lgals1^−/−^* macrophages infected with Ye wt or Ye ∆*yopP*,and found no significant changes in its synthesis (Figure 5C) Then, we analyzed NO production by WT or *Lgals1^−/−^* peritoneal macrophages after in vitro Ye infection.Remarkably, Gal1 and YopP induced a substantial regulation of NO and urea production (Figure 5D,E; *p* < 0.05). However, no significant differences in apoptosis were detected between WT and *Lgals1^−/−^* macrophages infected in vitro with Ye ∆*yopP* or Ye wt (Figure 5F).

To investigate possible mechanisms underlying this immunomodulatory effect, we inhibited ERK1/2 or p38 signaling pathways and then infected macrophages with Ye wt or Ye ∆*yopP*.Ye-driven suppression of NO synthesis was significantly prevented when both signaling pathways were interrupted (Figure 5G, *p* < 0.05). To evaluate whether the lack of Gal1 and/or YopP influences the clearance of Ye, we assessed bacterial load in PPs of WT or *Lgals1^−/−^* mice after 5 days of infection with Ye wt or with Ye ∆*yopP*. Significantly lower numbers of CFU were detected in PPs of Ye ∆*yopP* infected *Lgals1^−/−^* mice (Figure 5H, *p* < 0.01).

To confirm the effect of Ye ∆*yopP* infection and Gal1 on NO, iNOS expression was evaluated by Western blot. We observed inhibition of iNOS expression when WT macrophages were infected with Ye wt. On the contrary, an increased expression of iNOS was detected when *Lgals1^−/−^* macrophages were infected with Ye wt and Ye ∆*yopP* (Figure 5I, *p* < 0.05).

### 3.6. Exogenous Supplementation of rGal1 Does Not Influence the Protective Anti-Y. enterocolitica Response Observed in the Absence of YopP

**Figure 6:**
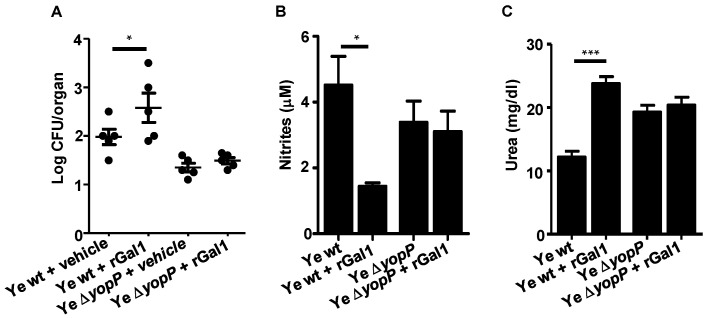
Exogenous Supplementation of rGal1 does not Revert the Protective Anti–Ye Response Observed in the Absence of YopP. *Lgals1*^−/−^ mice were treated i.p. with rGal1 or vehicle control daily for 5 d, starting on the day of infection. Mice were euthanized at day 5, and CFU were determined in homogenates of PPs. Limit of detectable CFU was 25 (log_10_25 = 1.4) (**A**). *Lgals1^−/−^* macrophages were infected with Ye wt or Ye ∆*yopP* for 1 h in the presence or absence of rGal1. NO production was determined in supernatants using Griess assay (**B**). Urea was determined in culture supernatants as an indirect evaluation of arginase activity (**C**). Data are representative of two independent experiments (*n* = 5 mice per group) (**A**). Data are mean ± SEM of two independent experiments (**B**,**C**). * *p* < 0.05, *** *p* < 0.001.

To evaluate the role of Gal1 and YopP in hindering anti-*Y. enterocolitica* immunity, we explored whether exogenous rGal1 could override the protective effect observed in *Lgals1^−/−^* hosts infected with Ye ∆*yopP*. In this regard, we have previously demonstrated that administration of exogenous rGal1 in Ye wt-infected *Lgals1^−/−^* mice abolished protection compared with untreated control *Lgals1^−/−^* mice [32]. However, the administration of rGal1 to *Lgals1^−/−^* mice infected with Ye ∆*yopP* showed a similar CFU number in PPs (Figure 6A), and no significant differences were observed in both NO and urea production when compared with the control group (Figure 6B,C), suggesting that the exogenous lectin does not restore the phenotype generated by Gal1 and/or YopP deficiency.

## 4. Discussion

Ye are Gram-negative bacteria that invade the intestine and use the type III protein secretion machinery to deliver bacterial effector proteins to host cells [2,4]. Similar to other microbes, the mechanisms underlying infection and immune evasion processes may involve bacterial glycoproteins recognized by host lectins, [36,43]. Several innate and adaptive immune cells, including macrophages, dendritic cells (DCs), and activated B and T cells, are an important source of Gal1 secretion [52,68,69,70]. In turn, this endogenous lectin controls the magnitude and nature of immune responses through diverse mechanisms including modulation of M1-M2 macrophage polarization, DC immunogenicity, regulatory T (Treg) cell expansion, T helper cell differentiation, and apoptosis [51,70,71,72,73,74,75,76]. Interestingly, Gal1 and its glycosylated ligands could be potentially used by pathogens as a glyco-checkpoint to subvert innate and adaptive immune programs [77]. In this sense, bacterial proteins such as Chlamydial membrane proteins MOMP and OmcB showed a permissive glycosylation pattern for Gal1 binding [43], and Gal-1 expressed by human cervical epithelial cells binds to the virulence factor lipophosphoglycan of *Trichomonas vaginalis* in a carbohydrate-dependent manner [78]. Additionally, Nita-Lazar et al. showed that upon influenza infection, *Streptococcus pneumoniae* adhesion to the airway epithelial surface is enhanced via the coordinated action of host galectins and viral and pneumococcal neuraminidases [79]. In this study, we provide evidence that Yops-secreted proteins from Ye- may bind Gal1 through carbohydrate-dependent mechanisms. Even though data on Ye glycosylation are still scarce, our findings showed the presence of permissive glycoepitopes for Gal1 binding in Yops, and particularly the relevant role of YopP in Gal1 binding to Ye, as demonstrated in binding experiments with Ye Δ*yopP*. Although alternative proteins, other than Yops, could be secreted from Ye [80], optimal culture conditions are offered for Yops secretion, among them, the addition of EGTA (5 mM) for Ca^2+^ chelation, MgCl_2_ (15 mM), and glucose (0.2%) [46]. Under these conditions, Yops represent a major component of the Ye secretome [81]. In this sense, a mass spectrometry-based identification of YopP showed several potentially glycosylated peptides; however, the poor number of b and y ion series during fragmentation, as well as low signal-to-noise ratio, hampered their full characterization. Further structural studies using a pre-enrichment technique and other strategies to improve the detection and analysis of glycopeptides would be relevant for a more complete understanding of Ye glycosylation pathways. Additionally, immunoprecipitation would also be useful to specifically verify these interactions.

Interestingly, we previously demonstrated that YopP up-regulates Gal1 expression in mouse splenocytes [32]. Here we found that Gal1 binds to the Ye surface and that lack of the critical effector protein YopP disrupts this association, highlighting specific interactions between YopP and Gal1. Although the functional role of Gal1-YopP interactions is unknown, previous studies showed a scaffold role for this lectin in other cell systems [82,83]. In this sense, we found that Gal1 prevents trypsin degradation of Yops. This finding is consistent with previous results demonstrating the biological relevance of this lectin in resistance to trypsin [84] and elastase [65] digestion. In this regard, using the software PeptideCutter (ExPASy), we identified several cleavage sites for trypsin on YopP and other Yops sequence (data not shown). Thus, Gal1 binding to Yops could represent an evolutionarily conserved mechanism to render bacterial virulence factors resistant to proteases implicated in infection.

It has been well established that macrophages confer early protection during the course of Ye infection [85]. NO synthesized by inducible iNOS is a major effector pathway of inflammatory macrophages; this inflammatory mediator plays essential roles in anti-microbial responses and host defense. Arginase catalyzes the alternative arginine metabolic pathway, which converts arginine to ornithine and urea [86]. Gal1 regulates L-arginine metabolism in peritoneal macrophages and microglia in this fashion by shifting the balance from classically-activated M1-type toward alternatively-activated M2-type macrophages and microglia [51,76].On the other hand, previous studies suggested that Yops corresponding to pathogenic *Yersinia* spp. inhibit LPS-mediated production of NO by macrophages [30]. Likewise, in the present study we found that YopP inhibited NO production and increased urea levels in a coordinate fashion with Gal1. In agreement with our findings, Silva Monnazzi et al. and Tansini et al. demonstrated that NO production in murine macrophages is suppressed by *Y. pseudotuberculosis* and that the YopP counterpart, YopJ, could be, at least in part, responsible of such effect [87,88]. In addition, we observed increased NO production by WT macrophages after inhibition of p38 and ERK1/2 signaling pathways and subsequent infection with Ye wt or Ye ∆*yopP*. These results are in agreement with our previous results showing that Gal1 production is regulated, at least in part, through p38 and ERK1/2 signaling pathways [32]. Although high levels of nitrogen species can damage basic cellular components and trigger cell death in macrophages [89], no significant differences in apoptosis were detected in *Lgals1^−/−^* peritoneal macrophages infected with Ye ∆*yopP* compared with the WT counterparts. In this regard, NO is a multifaceted molecule with dichotomous regulatory functions. Whereas it promotes apoptosis in several cell types, it prevents execution of cell death programs in other settings through specific inhibition of caspases [90].

Interestingly, Boland et al. (1998) showed that YopP inhibits TNF release by infected macrophages. Moreover, Giordano et al. (2011) [91] reported increased expression of pro-inflammatory cytokines, including TNF, in iNOS deficient innate immune cells. These results indicate that NO can inhibit the production of pro-inflammatory cytokines, which are usually produced by M1 macrophages [92] These data are in agreement with the unaltered TNF levels in absence of YopP, which could be due to the increased amounts of NO production.

A remarkable feature of macrophages is their plasticity. The classically-activated proinflammatory M1-type macrophages constitute one end of the spectrum, while alternatively activated anti-inflammatory M2 macrophages are on the other. Pro-inflammatory cytokines and mediators such as TNF and ROS are synthesized by M1 macrophages while anti-inflammatory factors such as IL-10, TGF-β, and arginase are considerably expressed in M2 macrophages [93]. To determine if a pro- or anti-inflammatory condition prevails in the presence of Gal1, we evaluate iNOS expression and IL-10 production in macrophages. We observed a key role of Gal1 in the negative modulation of iNOS expression, this finding is in agreement with those obtained by Starossom et al. (2012) who demonstrated that expression of iNOS mRNA was significantly decreased by Gal1 in M1 mice microglia [76]. In future studies, it would be useful to evaluated the polarization and inflammatory state of macrophages by monitoring their gene expression profile. On the other hand, it is well-known that IL-10 inhibits macrophage function and controls inflammation [92]. Moreover, several results showed that apoptotic macrophages trigger production of IL-10 [20,22]. We demonstrated that rGal1 supplementation restored NF-kB activation. TNF synthesis, and IL-6 production in PPs from *Lgals-1^−/−^* mice to levels comparable to those attained in WT hosts [32]. Here, we observed that administration of exogenous rGal1 to *Lgals-1*^−/−^ macrophages infected with Ye ∆*yopP* was not sufficient to restore decreased NO production and increased urea levels. Moreover, we observed that administration of exogenous Gal1 did not thwart the antibacterial protective effect unleashed in the absence of endogenous Gal1 and YopP. These results suggest that Gal1 and the bacterial virulence factor, YopP, might be crucial to regulate Ye pathogenesis using a coordinated mechanism, as has been reported for YopJ and IKKβ, MKK1, MKK2, MKK3, MKK4, MKK5, MKK6 [16,94] and IP6 [19]. Thus, in response to Ye infection, Gal1 and the virulence factor YopP may limit anti-bacterial responses. Conversely, deficiency in YopP or Gal1 controls the clearance of Ye and increases NO production. Additionally, our results suggest that ERK1/2 and p38 pathways mediate inhibition of NO production driven by Ye through mechanisms that could potentially involve regulation of Gal1 expression [95].

Thus, host derived Gal1 and glycosylated ligands may contribute to Ye infection by associating with YopP. In this regard, our studies identify glycosylation-dependent interactions between endogenous Gal1 and Yops that may play an important role during Ye infection through modulation of NO production. These findings may have critical implications in the design of tailored therapies aimed at controlling anti-bacterial responses during Ye infection. However, in spite of considerable progress, the clinical implications of our findings as well as the molecular mechanisms underlying YopP-Gal-1 interactions remain to be further investigated.

## Figures and Tables

**Table 1:** Lectins used for characterization of carbohydrate structures present in Yops.

Lectin	Ligands Described	Reference
*Arachis Hypogaea* (Peanut agglutinin) (PNA)	Galβ(1–3)GalNAc Galβ(1–3)GlcNAc Galβ(1–4)GlcNAc Lactose Galactose	[55]
*Erythrina crystagalli* (ECA)	Galβ(1–4)GlcNAc Lactose > GalNAc > Gal	[56]

**Table 2:** YopP peptides identified through nLC-MS/MS analysis.

Observed ^a^	Mass Expt ^b^	Mass (Theor) ^c^	Delta Error (Da) ^d^	Pep_exp_z ^e^	Start ^f^	End ^g^	Peptide Sequence ^h^	Modifications ^i^
707.816	2120.4261	2120.0208	0.4053	+3	232	250	**LNEYLNTNPQGVGTVVNKK**	Deamidated (NQ); Lys->CamCys (K)
1123.8687	3368.5843	3367.5	1.0843	+3	100	122	**FIINMGEGGIHFSVIDYKHINGK**	Deamidated (NQ); Lys->CamCys (K); Hex(1)HexNAc(1) (N); Hex(1)HexNAc(1) (ST); Oxidation (M)
1344.4664	2686.9182	2686.2353	0.6829	+2	48	67	**NYSRLDIEVMPALVIQANNK**	Deamidated (NQ); Hex(1)HexNAc(1) (N); Lys->CamCys (K)
954.9102	2861.7088	2861.3441	0.3647	+3	258	276	**FDNNKSIIDGKELSVSVHK**	Deamidated (NQ); 2 Hex(1)HexNAc(1) (ST)
470.2974	938.5802	937.5219	1.0583	+2	284	288	**TLLKV**	Hex(1)HexNAc(1) (ST)
604.0027	1205.9908	1205.5122	0.4787	+2	182	187	**KLYTER**	Hex(1)HexNAc(1) (ST); Lys->CamCys (K)
559.2594	1674.7563	1673.6991	1.0572	+3	250	262	**KNETIFNRFDNNK**	Deamidated (NQ); Lys->CamCys (K)
599.2961	1794.8664	1793.7499	1.1165	+3	21	29	**SLISNEELK**	Hex(1)HexNAc(1) (N); Hex(1)HexNAc(1) (ST); Lys->CamCys (K)
761.4185	1520.8224	1520.7471	0.0754	+2	200	213	**GILSDSENPLPHNK**	Deamidated (NQ)
515.7982	1544.3728	1544.6201	−0.2473	+3	251	262	**NETIFNRFDNNK**	Deamidated (NQ); Lys->CamCys (K)
1092.9822	3275.9248	3275.4749	0.4499	+3	1	20	**MIGPISQINSFGGLSEKETR**	Deamidated (NQ); Hex(1)HexNAc(1) (N); 2 Hex(1)HexNAc(1) (ST); Oxidation (M)
525.9216	1574.7429	1574.7069	0.036	+3	263	276	**SIIDGKELSVSVHK**	Lys->CamCys (K)
525.2371	2621.1492	2620.2921	0.8571	+5	30	51	**NIIIQLETDIADGSWFHKNYSR**	Deamidated (NQ)
890.7302	3558.8918	3558.7498	0.142	+4	1	29	**MIGPISQINSFGGLSEKETRSLISNEELK**	Deamidated (NQ); Hex(1)HexNAc(1) (N); Oxidation (M)
532.2823	1062.55	1062.5193	0.0307	+2	225	230	**HTQGKK**	Hex(1)HexNAc(1) (ST)
533.9495	1598.8266	1598.8767	−0.05	+1	278	288	**RIAEYKTLLK**	Hex(1)HexNAc(1) (ST)
793.7171	2378.1295	2378.0999	0.0296	+2	2	17	**IGPISQINSFGGLSEK**	Deamidated (NQ); 2 Hex(1)HexNAc(1) (ST)
642.8429	1283.6713	1282.4918	1.1795	+3	225	230	**HTQGKKR**	Hex(1)HexNAc(1) (ST); 2 Lys->CamCys (K)
793.4021	2377.1845	2376.9625	0.2219	+3	258	268	**FDNNKSIIDGK**	Hex(1)HexNAc(1) (N); Hex(1)HexNAc(1) (ST); Lys->CamCys (K)

^a^ The *m*/*z* (mass to charge state ratio) observed at the mass spectrometer, ^b^ The experimental mass of the peptide measured at the mass spectrometer, ^c^ The theoretical mass of the peptide obtained from the data base after in silico digestion, ^d^ The error of the peptide mass in Da calculated from theoretical mass minus experimental mass obtained from the mass spectrometry analysis, ^e^ Peptide charge state after nanoESI ionization, ^f^ Position of the first residue of the peptide identified in the whole protein sequence, ^g^ Position of the last residue of the peptide identified in the whole protein sequence, ^h^ Peptide sequence retrieved from the protein database after the bioinformatics analysis, ^i^ Potential modifications observed at the peptide identified.
